# Threshold Computation for Spatially Coupled Turbo-Like Codes on the AWGN Channel

**DOI:** 10.3390/e23020240

**Published:** 2021-02-19

**Authors:** Muhammad Umar Farooq, Alexandre Graell i Amat, Michael Lentmaier

**Affiliations:** 1 Department of Electrical and Information Technology, Lund University, 22100 Lund, Sweden; michael.lentmaier@eit.lth.se; 2 Department of Electrical Engineering, Chalmers University of Technology, 41296 Gothenburg, Sweden; alexandre.graell@chalmers.se

**Keywords:** spatially coupled codes, turbo codes, concatenated convolutional codes, density evolution, iterative decoding, coding thresholds, AWGN channel

## Abstract

In this paper, we perform a belief propagation (BP) decoding threshold analysis of spatially coupled (SC) turbo-like codes (TCs) (SC-TCs) on the additive white Gaussian noise (AWGN) channel. We review Monte-Carlo density evolution (MC-DE) and efficient prediction methods, which determine the BP thresholds of SC-TCs over the AWGN channel. We demonstrate that instead of performing time-consuming MC-DE computations, the BP threshold of SC-TCs over the AWGN channel can be predicted very efficiently from their binary erasure channel (BEC) thresholds. From threshold results, we conjecture that the similarity of MC-DE and predicted thresholds is related to the threshold saturation capability as well as capacity-approaching maximum a posteriori (MAP) performance of an SC-TC ensemble.

## 1. Introduction

Turbo-like codes (TCs) [1]—such as parallel concatenated codes (PCCs) and serially concatenated codes (SCCs)—and low-density parity-check (LDPC) codes [2] are widely used in communication systems due to their excellent performance and low-complexity decoding. In most cases, the design of these codes is based on the optimization of the iterative belief propagation (BP) decoding threshold, which can be performed via density evolution (DE).

The exact BP thresholds of LDPC codes over the binary erasure channel (BEC) can be easily obtained recursively from a set of DE equations using a scalar representation of the message densities, whereas for the AWGN channel they can be obtained via quantized DE [3]. Alternatively, the BP threshold may be estimated by means of extrinsic information transfer (EXIT) function analysis [4,5], where the densities of the messages are approximated by a Gaussian distribution. For the AWGN channel and binary transmission, the Gaussian approximation yields thresholds close to those obtained via DE, while simplifying the computation. The BP thresholds of the major TC ensembles—PCCs, SCCs, braided convolutional codes (BCCs), and hybrid concatenated codes (HCCs)—over the BEC were computed by Moloudi et al. in [6,7] by using the decoder transfer functions of the component codes that map the input and output erasure probabilities of the message sequences. Unfortunately, the decoder transfer functions are not available for the AWGN channel, which hinders the derivation of the exact DE equations. In [8], a Monte Carlo (MC)-based DE (MC-DE) was proposed for the threshold analysis of BCCs over the AWGN channel. The MC-DE, however, is computationally demanding compared to the simple DE for TCs over the BEC. Moreover, MC-DE for TCs, which entails running BCJR decoding of the component codes, is significantly harder than the quantized DE or the EXIT chart technique for LDPC codes.

Spatial coupling [9] allows us to construct particularly powerful codes. Thanks to the threshold saturation phenomenon, the BP decoding threshold of a spatially coupled ensemble can achieve the maximum a posteriori (MAP) decoding threshold of the underlying uncoupled ensemble. Remarkably, spatially coupled LDPC codes universally achieve capacity over the class of binary-input memoryless symmetric channels [10]. The concept of spatial coupling was extended to turbo-like codes in [6].

Quantized DE for SC-LDPC codes is time consuming, due to the large number of edge types in the corresponding graph. The complexity of MC-DE of spatially coupled (SC) TCs (SC-TCs) is even higher, and hence the computation of the thresholds becomes challenging. Efficient methods that allow to accurately predict the BP thresholds are therefore of practical interest. In [11], the BP thresholds of randomly-punctured LDPC codes over the AWGN channel were efficiently predicted by using their corresponding BEC thresholds. The same idea was later used in [8] to predict the thresholds of BCCs over the AWGN channel using the BEC thresholds of BCCs [6]. The resulting thresholds for SC-BCCs, which have a regular graph representation, are close to those obtained using MC-DE, which can be attributed to the universality of this ensemble.

In this paper, we perform a comprehensive threshold analysis of several classes of uncoupled and coupled TCs—PCCs, SCCs, BCCs, and HCCs—over the AWGN channel, by discussing several efficient methods for threshold computation. More concretely, we review MC-DE with Gaussian approximation and MC-DE where the true densities are estimated using histograms. We also discuss the prediction of the BP thresholds using the thresholds of the corresponding ensembles over the BEC. Further, we discuss the efficient computation of the BP thresholds of punctured TCs from those of the corresponding mother code. We show that for spatially coupled TC ensembles with strong underlying uncoupled code, a very accurate prediction of the BP threshold over the AWGN channel can be efficiently obtained for a large range of coding rates from the BP threshold of the corresponding mother code ensembles over the BEC. We conjecture that the accurate predictions can be attributed to the universality of these code ensembles due to threshold saturation.

The rest of the paper is structured as follows. In Section 2, the construction of uncoupled and coupled TC ensembles is described using uncoupled and coupled SCCs as an example. A base matrix representation is introduced, which is then used to define the remaining ensembles. In Section 3, MC-DE for the computation of the BP thresholds of TCs is described in detail. In Section 4, we discuss threshold prediction methods for randomly punctured TCs. In Section 5, we compare and discuss the thresholds computed via the different methods for several classes of uncoupled and coupled TCs. Finally, Section 6 concludes this work.

## 2. Preliminaries

We consider the TC ensembles in [6,7] with and without coupling. In this section, we describe these ensembles by discussing SCCs and SC-SCCs of coupling memory m=1 and refer the reader to [6] and [7] for detailed description of other TCs. In order to efficiently describe the coupling for each of the ensembles, we introduce a new base matrix representation corresponding to the compact graph representation of the ensembles in [6]. Lastly, we briefly describe the sliding window decoder, which is used in this work to carry out the threshold analysis.

### 2.1. Code Ensembles

**Figure 1:**
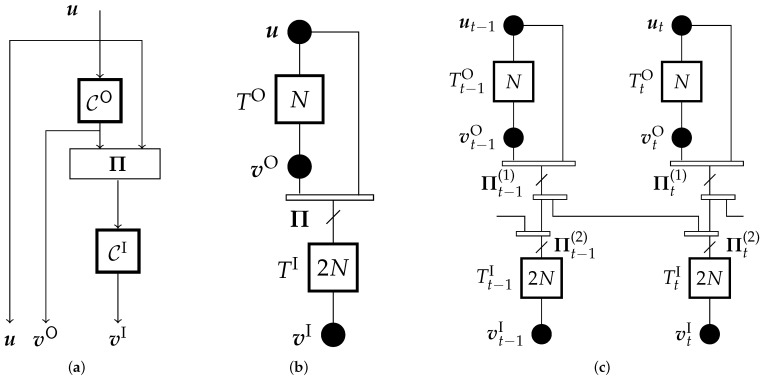
(**a**) Encoder block diagram of SCC, (**b**) Compact graph representation of SCC Encoder, (**c**) SC-SCC.

The block diagram of a rate-1/4 SCC encoder is shown in Figure 1a. The encoder is constructed from two recursive systematic convolutional encoders, referred to as outer and inner encoders. The information sequence u is first encoded by the outer encoder $C^{O}$, resulting in the encoded sequence $v^{O}$. The sequence $\left(u,v^{O}\right)$ is reordered by a permuter Π and then encoded by the inner encoder $C^{I}$, producing the parity sequence $v^{I}$. The codeword sequence $v=\left(u,v^{O},v^{I}\right)$ is obtained at the output of the SCC encoder after following these encoding steps.

The compact graph of the SCC ensemble is shown in Figure 1b. The sequences u, $v^{O}$ and $v^{I}$ are represented by the black circles in the compact graph, which are referred to as variable nodes, and the trellises corresponding to $C^{O}$ and $C^{I}$ are represented by squares, referred to as constraint nodes. Each constraint node is labeled with the corresponding trellis length. The sequences u and $v^{O}$ are connected to the outer constraint node $T^{O}$. Similarly, the sequence $\left(u,v^{O}\right)$, permuted through a permuter Π, and the sequence $v^{I}$ are connected to the inner constraint node $T^{I}$. The sequence $\left(u,v^{O}\right)$ in the compact graph is obtained by a multiplexer, which is indicated by the rectangle. The permuter Π is shown as a line that crosses the edge connecting the inner constraint node with the multiplexer.

Figure 1c shows the compact graph of the spatially coupled SCC (SC-SCC) ensemble with coupling memory m=1 at time *t*. Consider a collection of SCC compact graphs at times t=1,…,L, where *L* denotes the coupling length. Denote by $s_{t}$ the sequence $\left(u_{t},v_{t}^{O}\right)$ and by ${\tilde{s}}_{t}$ the reordered sequence, reordered by permutation $\Pi_{1}$. The SC-SCC ensemble is constructed by dividing the sequence ${\tilde{s}}_{t}$ into two sub-sequences, denoted as ${\tilde{s}}_{t,k}$ for k=0,1, and spreading them over times *t* and t+1. The sequence $\left({\tilde{s}}_{t,0},{\tilde{s}}_{t-1,1}\right)$ at the input of $T_{t}^{I}$ is permuted by permuter $\Pi_{2}$ before producing the parity sequence $v_{t}^{I}$. The information bits at time t≤0 are initialized to zero.

### 2.2. Representation of Spatially Coupled Turbo-Like Codes

We introduce a base-matrix representation corresponding to the compact graphs of TC ensembles, similar to that of protograph LDPC codes. Starting with the SCC ensemble in Figure 1, we define for each ensemble a connection matrix P, which is the bi-adjacency matrix of the lifted compact graph. From P, the base matrices of the coupled and uncoupled ensembles can be identified.

The outer constraint node $T^{O}$ of the SCC in Figure 1b is connected to $v^{O}$ and u, both representing *N* bits, as indicated by the label of the constraint node. These connections are represented in the first row of a connection matrix $P_{\mathrm{SCC}}$ by the two N×N identity matrices $I_{N}$. The edges from $v^{O}$ and u are first merged and then connected to the inner constraint node $T^{I}$, after permutation by Π. This is represented by the matrix $P_{2N}=\Pi$ of size 2N×2N in the second row of $P_{\mathrm{SCC}}$. Similarly, the connections along the edge of variable node $v^{I}$—representing 2N bits—to $T^{I}$ is captured by the identity matrix $I_{2N}$.

(1) $$\begin{array}{ccc} & \mathrm{node} & \begin{array}{cccc} u\;\; & & v^{O} & v^{I} \end{array}\\ P_{\mathrm{SCC}}= & \begin{array}{c} T^{O}\\ T^{I} \end{array} & [\begin{array}{cccc} I_{N} & & I_{N} & 0\\ & P_{2N} & & I_{2N} \end{array}]. \end{array}$$

The connection matrix representation allows to describe the ensemble in terms of a base matrix, analogous to the base matrix of a protograph-based LDPC code. In the base matrix, the individual permutation matrices in the connection matrix are replaced by a 1 and the zero matrices by a 0, resulting in

$$\begin{array}{c} B \end{array}=\left[\begin{array}{cccc} 1 & \circ & 1 & 0\\ \circ & 1 & \circ & 1 \end{array}\right].$$

The base matrix represents an ensemble of codes, defined by the set of possible permutation matrices that can be used in the lifting procedure. In order to lift the base matrix B to a particular connection matrix $P_{\mathrm{SCC}}$, each 1 is replaced by a permutation matrix and each 0 by an all-zero matrix. Note that the matrices have different sizes, which can be identified from the connection matrix (1). The entries denoted by ∘ are placeholders that are required because of the merging of two edges of width *N* into one edge of width 2N in the compact graph. To make our notation consistent, in the lifting procedure we replace each ∘ in the base matrix by an empty matrix with column dimension zero. There is a one-to-one correspondence between the base matrix and the compact graph representation provided that the lengths of the component encoder trellises are known.

A coupled ensemble can be obtained by partitioning B into submatrices $B_{i}$ such that $B=\sum_{i=0}^{m}B_{i}$ [12]. For the ensemble in Figure 1c, we get

$$\begin{array}{c} B_{0} \end{array}=\left[\begin{array}{cccc} 1 & \circ & 1 & 0\\ \circ & \frac{1}{m+1} & \circ & 1 \end{array}\right],\begin{array}{c} B_{i>0} \end{array}=\left[\begin{array}{cccc} 0 & \circ & 0 & 0\\ \circ & \frac{1}{m+1} & \circ & 0 \end{array}\right].$$

The fraction $\frac{1}{m+1}$ in $B_{i}$ indicates that $\frac{1}{m+1}\cdot 2N$ bits out of the 2N bits represented by the variable nodes of the SC-SCC graph at time *t* are connected with the trellises at time t+i in a randomized way.

Following the same procedure, the connection matrix of uncoupled BCCs and the base matrices of SC-BCCs are obtained as

$$\begin{array}{ccc} & \mathrm{node} & \begin{array}{ccc} u\; & v^{U}\; & v^{L} \end{array}\\ P_{\mathrm{BCC}}= & \begin{array}{c} T^{U}\\ T^{L} \end{array} & [\begin{array}{ccc} I_{N} & I_{N} & P_{N}^{U}\\ P_{N} & P_{N}^{L} & I_{N} \end{array}], \end{array}\;B_{0}=\left[\begin{array}{ccc} 1 & 1 & 0\\ 1 & 0 & 1 \end{array}\right],\;B_{i>0}=\left[\begin{array}{ccc} 0 & 0 & \frac{1}{m}\\ 0 & \frac{1}{m} & 0 \end{array}\right].$$

and for PCCs as

$$\begin{array}{ccc} & \mathrm{node} & \begin{array}{ccc} u\; & v^{U} & v^{L} \end{array}\\ P_{\mathrm{PCC}}= & \begin{array}{c} T^{U}\\ T^{L} \end{array} & [\begin{array}{ccc} I_{N} & I_{N} & 0\\ P_{N} & 0 & I_{N} \end{array}], \end{array}\;B_{0}=\left[\begin{array}{ccc} \frac{1}{m} & 1 & 0\\ \frac{1}{m} & 0 & 1 \end{array}\right],\;B_{i>0}=\left[\begin{array}{ccc} \frac{1}{m} & 0 & 0\\ \frac{1}{m} & 0 & 0 \end{array}\right].$$

The connection matrix of the uncoupled HCC ensemble is

$$\begin{array}{ccc} & \mathrm{node} & \begin{array}{cccccc} u & & v^{U}\;\; & & v^{L}\; & v^{I} \end{array}\\ P_{\mathrm{HCC}}= & \begin{array}{c} T^{U}\\ T^{L}\\ T^{I} \end{array} & [\begin{array}{ccccc} I_{N} & I_{N} & & 0 & 0\\ P_{N} & 0 & & I_{N} & 0\\ 0 & & P_{2N}^{1} & & I_{2N} \end{array}]. \end{array}$$

In [7], two spatially coupled ensembles of HCCs, referred to as Type-I SC-HCCs and Type-II SC-HCCs, were introduced. For Type-I, the base matrices are

$$\begin{array}{c} B_{0} \end{array}=\left[\begin{array}{ccccc} 1 & 1 & \circ & 0 & 0\\ 1 & 0 & \circ & 1 & 0\\ 0 & \circ & \frac{1}{m+1} & \circ & 1 \end{array}\right],\begin{array}{c} B_{i>0} \end{array}=\left[\begin{array}{ccccc} 0 & 0 & \circ & 0 & 0\\ 0 & 0 & \circ & 0 & 0\\ 0 & \circ & \frac{1}{m+1} & \circ & 0 \end{array}\right],$$

whereas for Type-II are obtained as

$$\begin{array}{c} B_{0} \end{array}=\left[\begin{array}{ccccc} \frac{1}{m+1} & 1 & \circ & 0 & 0\\ \frac{1}{m+1} & 0 & \circ & 1 & 0\\ 0 & \circ & \frac{1}{m+1} & \circ & 1 \end{array}\right],\begin{array}{c} B_{i>0} \end{array}=\left[\begin{array}{ccccc} \frac{1}{m+1} & 0 & \circ & 0 & 0\\ \frac{1}{m+1} & 0 & \circ & 0 & 0\\ 0 & \circ & \frac{1}{m+1} & \circ & 0 \end{array}\right].$$

Throughout this paper we consider convolutional encoders with four-state trellises in all threshold computations and finite length simulations. In particular, rate-2/3 encoders with generator matrix

$$G=\left[\begin{array}{ccc} 1 & 0 & 1/7\\ 0 & 1 & 5/7 \end{array}\right]$$

are used for BCCs and rate-1/2 encoders with generator matrix G=[15/7] are used for all other ensembles.

### 2.3. Sliding Window Decoding

In this work, for the threshold analysis we assume SC-TCs of coupling length L=∞ and coupling memory m=1 under sliding window decoding with on-demand symbol node updating schedule [9] of window size *W*. In this schedule, the constraint nodes within the window are updated sequentially by receiving the most recent updated messages from the neighboring nodes. Note that a larger window size is required for the computation of the thresholds of coupled ensembles with larger *m*, as the window size needs to be large enough so that the decoding wave is formed. All our threshold computations are carried out by considering W=20, which is observed to be enough for a reliable estimate of the decoding thresholds for the considered ensembles with m={1,3}, yet allowing an efficient computation. In our finite length simulations we use W=8.

## 3. Threshold Computation via Monte Carlo Density Evolution

The BP thresholds can be computed by performing DE. For codes for which the transfer functions of the component codes are not available, MC-DE can be used. In this section, we describe two MC-DE methods and discuss their advantages and shortcomings. The described MC-DE methods are computationally demanding. We hence also discuss an efficient method that provides an approximation of the BP threshold, and compare the thresholds obtained applying the three approaches for uncoupled SCCs.

### 3.1. Monte-Carlo Density Evolution

MC-DE comprises three key steps [8], which are performed a number of iterations:

1. Variable node update: A variable node generates a sequence of extrinsic log-likelihood ratios (LLRs) by properly combining the sequence of Gaussian-distributed channel LLRs and the sequences of extrinsic LLRs—generated according an appropriate distribution—received from the neighboring constraint nodes. In particular, the output LLRs are obtained as the sum of the channel LLR and the LLRs from neighboring constraint nodes.
2. Constraint node update: A constraint node performs BCJR decoding on the sequences of extrinsic LLRs—used as a-priori information—received from the neighboring variable nodes. The BCJR decoder generates sequences of extrinsic LLRs that are passed to the corresponding neighboring variable nodes.
3. Density estimation and re-sampling: A sequence of channel LLRs, $L_{\mathrm{ch}}$, and the sequences of extrinsic LLRs, $L_{\mathrm{ext}}$ (one for each code sequence), are created from the corresponding probability densities $f(L_{\mathrm{ch}})$ and $f(L_{\mathrm{ext}})$. These sequences are used in the next MC-DE iteration.

MC-DE tracks the evolution of $f(L_{\mathrm{ext}})$ through the iterative decoding process.

### 3.2. Monte-Carlo Density Evolution with Gaussian Approximation

In MC-DE with Gaussian approximation (MC-DE-GA), the densities $f(L_{\mathrm{ext}})$ are approximated by a Gaussian distribution, which can be characterized by its mean $m_{G}$ and standard deviation $\sigma_{}$. Since the extrinsic LLRs are symmetric and consistent, the Gaussian distribution is completely characterized by the single parameter σ, which is related to the mean $m_{G}$ as

(2) $$m_{G}=\frac{\sigma^{2}}{2}.$$

The standard deviation σ is computed from the mutual information $I_{E}$ between an extrinsic LLR sequence $L_{\mathrm{ext}}$ and the corresponding binary code sequence [4,5],

(3) $$\sigma =C_{G}^{-1}\left(I_{E}\right).$$

Here $C_{G}\left(\sigma \right)$ denotes the AWGN channel capacity for a given channel parameter σ, which can be computed efficiently using the following series expansion ([13] Chapter 4):

(4) $$C_{G}\left(\sigma \right)=1+\frac{1}{\ln2}\left(\left(\frac{2}{\sigma^{2}}-1\right)Q\left(\frac{1}{\sigma }\right)-\sqrt{\frac{2}{\pi \sigma^{2}}}e^{-\frac{1}{2\sigma^{2}}}+\sum_{i=1}^{\infty }\frac{{\left(-1\right)}^{i}}{i(i+1)}e^{\frac{2i(i+1)}{\sigma^{2}}}Q\left(\frac{1+2i}{\sigma }\right)\right)\;$$

with $Q\left(x\right)=\frac{1}{\sqrt{2\pi }}\int_{x}^{\infty }e^{-\frac{y^{2}}{2}}dy$. The mutual information $I_{E}$ is computed from [5]

(5) $$I_{E}\approx 1-\frac{1}{N^{\prime }}\sum_{n=1}^{N^{\prime }}\log_{2}\left(1+e^{-L_{\mathrm{ext},n}}\right),$$

where $L_{\mathrm{ext},n},n=1,\cdots ,N^{\prime }$, are the elements of $L_{\mathrm{ext}}$.

Note that MC-DE-GA is equivalent to an EXIT function analysis. While a threshold computation via the Gaussian assumption becomes highly efficient for LDPC codes, MC-DE-GA has only a minor computational advantage for TCs. Furthermore, for several ensembles, in particular multi-edge type ensembles such as BCCs, SCCs, HCCs, and SC-TCs, the true distributions of the messages may significantly deviate from a Gaussian distribution, leading to inexact decoding thresholds.

### 3.3. MC-DE with Histogram

A more accurate BP threshold can be obtained by estimating the true densities, which can be performed by means of histograms. We refer to this method as MC-DE-H, which we describe in detail in the following. For ease of notation, let *L* be the random variable corresponding to an extrinsic LLR. From the consistency property of the LLRs, we have that $f_{L}\left(l\right)=e^{l}\cdot f_{L}\left(-l\right)$ [5]. The consistency and symmetry properties of *L* allow us to determine $f_{L}\left(l\right)$ from the distribution of |L| from [13]

(6) $$f_{L}\left(l\right)=I_{\left\{l\geq 0\right\}}\frac{1}{1+e^{-l}}f_{\left|L\right|}\left(l\right)+I_{\left\{l\leq 0\right\}}\frac{e^{l}}{1+e^{l}}f_{\left|L\right|}\left(-l\right).$$

Taking advantage of this symmetry drastically improves the speed of converge of this method.

Without loss of generality, we consider the transmission of the all-zero codeword and approximate the density $f_{L}\left(l\right)$ in (6) with a histogram of *M* bins and obtain an approximated probability mass function $\hat{P}\left(l\right)$ (For the threshold calculations within this paper we use a fixed number of M=2001 bins, divided uniformly between $-L_{\max}$ and $+L_{\max}$, where $L_{\max}=\max_{n}\left|L_{n}\right|$ denotes the maximum magnitude among the elements $L_{n}$ of the measured sequence. An odd value of *M* is recommended to represent erasures accurately. The length of the sequence is chosen adaptively to achieve a desired accuracy). The bit error rate (BER) can be computed from $\hat{P}\left(l\right)$ as

(7) $$BER\approx \sum_{\left\{l<0\right\}}^{}\hat{P}\left(l\right).$$

We denote by ${\hat{f}}_{L}\left(l\right)$ the approximation of $f_{L}\left(l\right)$. A sequence of extrinsic LLRs distributed according to ${\hat{f}}_{L}\left(l\right)$ can be obtained from $\hat{P}\left(l\right)$ by using the probability integral transform. The probability integral transform method states that for a uniformly distributed random variable U∈[0,1], and a strictly increasing cumulative distribution function ${\hat{F}}_{L}\left(l\right)$, we have $U={\hat{F}}_{L}\left(l\right)\approx \hat{P}\left(L\leq l\right)$. Samples are generated from ${\hat{F}}_{L}\left(l\right)$ by applying the inversion ${\hat{F}}_{L}^{-1}\left(U\right)$.

### 3.4. Erasure Channel Prediction

Since MC-DE is time consuming, we are interested in exploring some faster alternatives to predict the BP thresholds of TCs over the AWGN channel. In [3], the erasure channel prediction (ECP) method was proposed to efficiently predict BP thresholds of codes over the AWGN channel from their corresponding BEC thresholds $\epsilon^{*}$. For a given code, the AWGN channel BP threshold $\sigma^{*}$ can be obtained from the corresponding $\epsilon^{*}$ as

(8) $$\sigma^{*}\approx C_{G}^{-1}\left(C_{E}\left(\varepsilon^{*}\right)\right)=C_{G}^{-1}\left(1-\varepsilon^{*}\right).$$

where $C_{E}\left(\epsilon^{*}\right)=1-\epsilon^{*}$ is the capacity of the BEC.

### 3.5. Discussion

In Table 1 we give the BP thresholds of the uncoupled SCCs computed via MC-DE-GA, MC-DE-H, and ECP, for several code rates. We observe that both MC-DE-GA and MC-DE-H yield similar thresholds. However, we remark that the computational complexity of MC-DE-GA and MC-DE-H is similar, as it is primarily dominated by that of the BCJR decoder. Hence, one may resort to MC-DE-H, which does not rely on a Gaussian assumption and gives a more accurate estimation of the threshold provided that the quantization resolution is chosen sufficiently high. The thresholds predicted by the ECP method differ noticeably from those predicted by the MC-DE methods.

## 4. Efficient AWGN Channel Threshold Predictions of Randomly Punctured TCs

Following the ideas in [11], efficient methods for predicting the thresholds of randomly punctured BCCs were investigated in [8], namely the $\theta_{E}$ prediction, the $\theta_{G}$ prediction, and the mixed prediction (MP) method. In this section, we re-visit these methods by analyzing the MC-DE-H and predicted thresholds of the SCC ensemble as an example.

### 4.1. $\theta_{E}$-Predictions

Consider a code ensemble of rate R(α) obtained by randomly puncturing a mother code of rate R=R/(1−α), where 0≤α<1 is the puncturing fraction, i.e., the fraction of bits that are punctured. For the BEC, the BP threshold of the punctured code ensemble, $\epsilon^{*}\left(\alpha \right)$, can be obtained as

(9) $$\epsilon^{*}\left(\alpha \right)=1-\theta_{E}R\left(\alpha \right)\;,$$

where

(10) $$\theta_{E}=\frac{1-\epsilon^{*}}{R},$$

with $\epsilon^{*}$ being the BP threshold of the mother code.

The BP threshold $\sigma^{*}\left(\alpha \right)$ of a randomly punctured ensemble over the AWGN channel can be predicted from the BEC threshold of the corresponding mother code by combining (9) with the ECP in (8) to

(11) $$h_{G}\left(\sigma^{*}\left(\alpha \right)\right)\approx h_{E}\left(\varepsilon^{*}\left(\alpha \right)\right)=\epsilon^{*}\left(\alpha \right)=1-\theta_{E}R\left(\alpha \right).$$

Here, $h_{G}\left(\sigma \right)=1-C_{G}\left(\sigma \right)$ and $h_{E}\left(\varepsilon \right)=1-C_{E}\left(\varepsilon \right)=\varepsilon$ denote the conditional entropy of the AWGN channel and the BEC, respectively. We refer to this method as $\theta_{E}$-prediction.

The $\theta_{E}$-predictions and the MC-DE-H thresholds of SCCs and SC-SCCs with m=1 and different code rates are shown in Table 2, where ϵ and $\epsilon_{\mathrm{SC}}$ denote the BEC thresholds of SCCs and SC-SCCs with m=1, respectively. It is observed that with coupling, the thresholds computed using the $\theta_{E}$-prediction are similar to those obtained via MC-DE-H, i.e., the $\theta_{E}$-prediction yields accurate thresholds. For SCCs, however, the thresholds differ noticeably. We remark that punctured bits can be equivalently seen as erasures. Hence, the behavior of the ensembles over the AWGN channel becomes closer to their behavior over the BEC for increasing puncturing fraction. This explains that the relative difference between the $\theta_{E}$-prediction and MC-DE-H thresholds is larger for lower rates. We conjecture that the accurateness of the $\theta_{E}$-prediction for the SC-SCC ensemble is due to the universality of this ensemble. Indeed, it was shown in [6] that for large-enough coupling memory, SC-SCCs approach capacity.

### 4.2. $\theta_{G}$-Predictions

The gap between the MC-DE-H threshold and the $\theta_{E}$-prediction for low rates can be reduced by using the $\theta_{G}$-prediction [8]. This prediction method uses the AWGN channel threshold of the mother code to determine the BP threshold of the punctured code. Using the $\theta_{G}$-prediction, the AWGN channel threshold $h_{G}\left(\sigma^{*}\right)$ is obtained as

(12) $$h_{G}\left(\sigma^{*}\left(\alpha \right)\right)\approx 1-\theta_{G}R\left(\alpha \right),$$

where

(13) $$\theta_{G}=\frac{1-h_{G}\left(\sigma^{*}\right)}{R}.$$

The $\theta_{G}$-predictions for SCCs are shown in Table 3. For low rates, the $\theta_{G}$-predictions are close to the MC-DE-H thresholds. However, the $\theta_{G}$-prediction fails to accurately predict the thresholds for higher rates, and a gap to the MC-DE-H thresholds is observed.

### 4.3. Mixed Predictions

**Figure 2:**
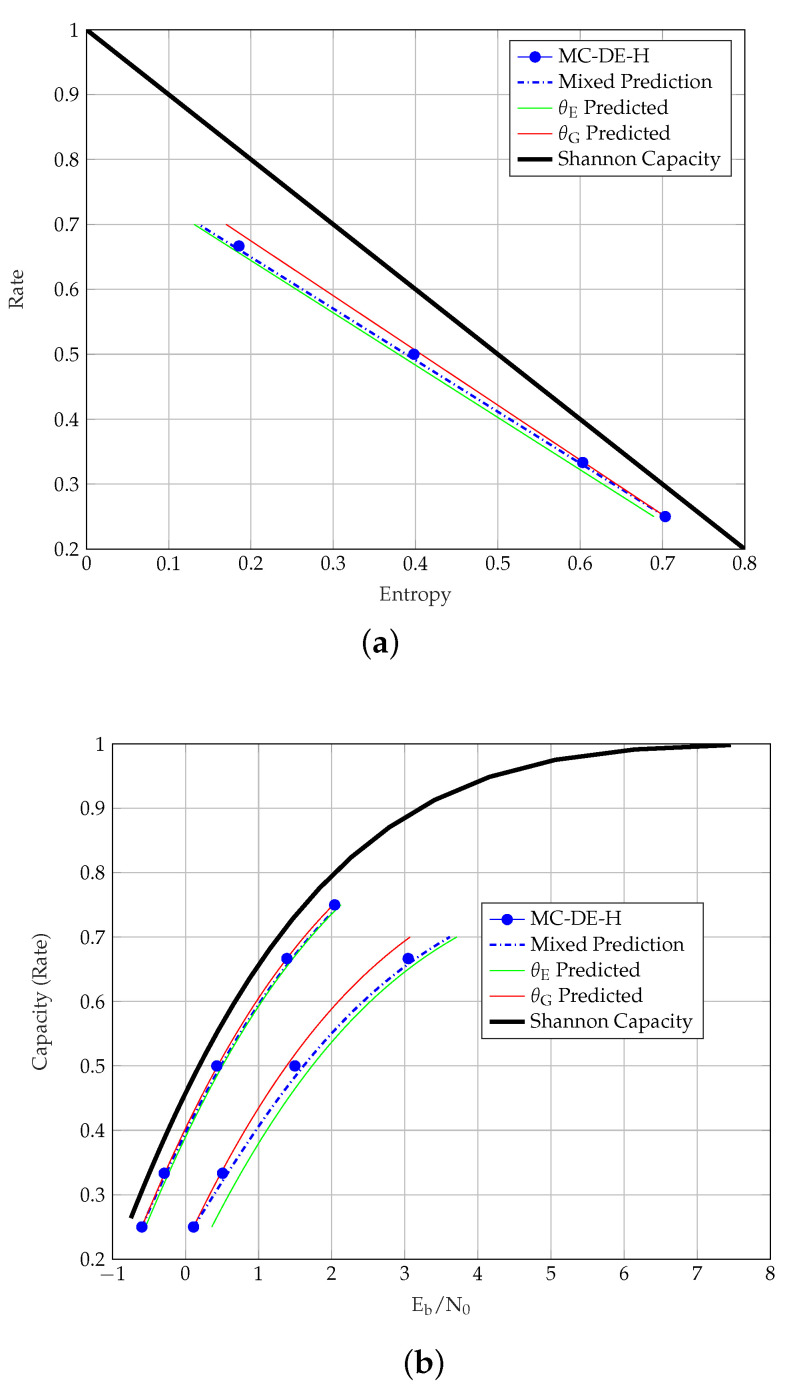
AWGN channel thresholds of uncoupled and coupled SCCs. (**a**) SCC Entropy vs. Capacity. (**b**) SCC and m=1 SC-SCC $E_{b}/N_{0}$ vs. Capacity.

A mixed prediction (MP) method is proposed in [8] to overcome the discrepancies observed in the $\theta_{E}$ and the $\theta_{G}$ predictions. The idea of the MP stems in [11], where the AWGN channel thresholds of randomly punctured LDPC codes were observed to lie on a straight line in the entropy perspective. The MP method uses both $\theta_{E}$ and $h_{G}\left(\sigma^{*}\right)$ in accurately predicting the thresholds of randomly punctured LDPC codes at all rates. For randomly punctured TCs, as shown for randomly punctured SCCs in Figure 2a, we can observe that the AWGN channel thresholds tend to follow a straight line as well. From $\theta_{E}$ and $h_{G}\left(\sigma^{*}\right)$, the predicted thresholds of the punctured TCs via the MP method [8] are obtained by using

(14) $$h_{G}\left(\sigma_{BP}\left(\alpha \right)\right)\approx h_{G}\left(\sigma^{*}\right)-\theta_{\mathrm{MP}}\left(R(\alpha )-R\right),$$

where $\theta_{\mathrm{MP}}$ is

(15) $$\theta_{\mathrm{MP}}=\frac{\theta_{E}+h_{G}\left(\sigma^{*}\right)-1}{1-R}.$$

The MP thresholds are shown as a dashed line in Figure 2a. It is observed that the SCC MP thresholds deviate slightly from the MC-DE-H thresholds at medium rates, unlike the more accurate MP thresholds for the LDPC codes in [11]. In fact, even for LDPC codes it is still an open problem to prove the conjecture that AWGN channel thresholds follow a straight line with random puncturing. For TC ensembles with more complicated component codes this is even harder to prove and may be wrong. On the other hand, the deviations we observe are small enough to use the MP thresholds as an efficient approximation.

The MC-DE-H and predicted thresholds of the uncoupled and coupled SCCs over the AWGN channel are shown in Figure 2b, where the MP method is observed to be a more suitable representative of the MC-DE-H thresholds for all the considered rates.

## 5. Threshold Comparison for Different TC Ensembles

**Figure 3:**
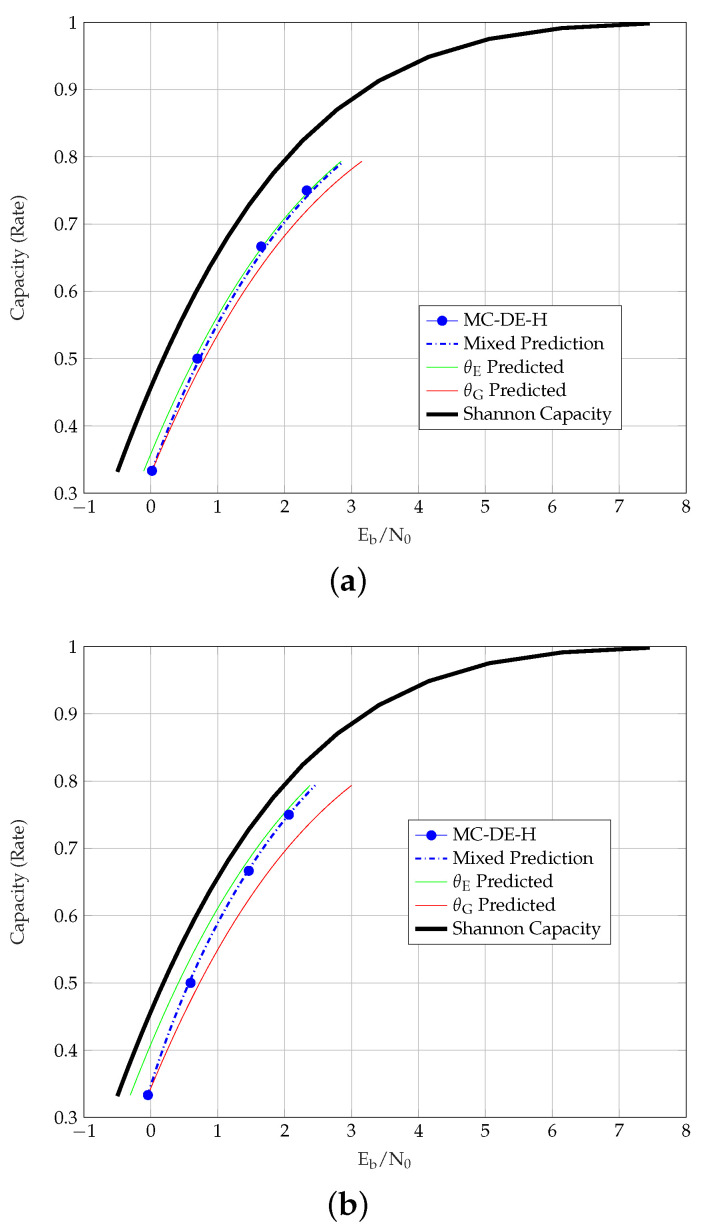
Thresholds of PCCs over the AWGN channel. (**a**) $E_{b}/N_{0}$ vs. Rate of PCC. (**b**) $E_{b}/N_{0}$ vs. Rate of m=1 SC-PCC.

Table 4 presents the MC-DE-H and $\theta_{E}$ predicted thresholds of the coupled TCs. Unlike uncoupled TCs, the predictions are observed to be relatively close to the MC-DE-H thresholds. For this reason, we do not list the $\theta_{G}$- or MP thresholds of SC-TCs in the table. For the uncoupled ensembles, however, the MP method provides better predicted thresholds than the $\theta_{E}$ method. We further observe that, in general, the $\theta_{G}$ predictions are closer to the Shannon capacity than the $\theta_{E}$ predictions. The exception to this are PCCs, where $\theta_{E}$ predictions are closer to the Shannon capacity than the $\theta_{G}$ predictions, and the gap increases with coupling, as shown in Figure 3.

**Figure 4:**
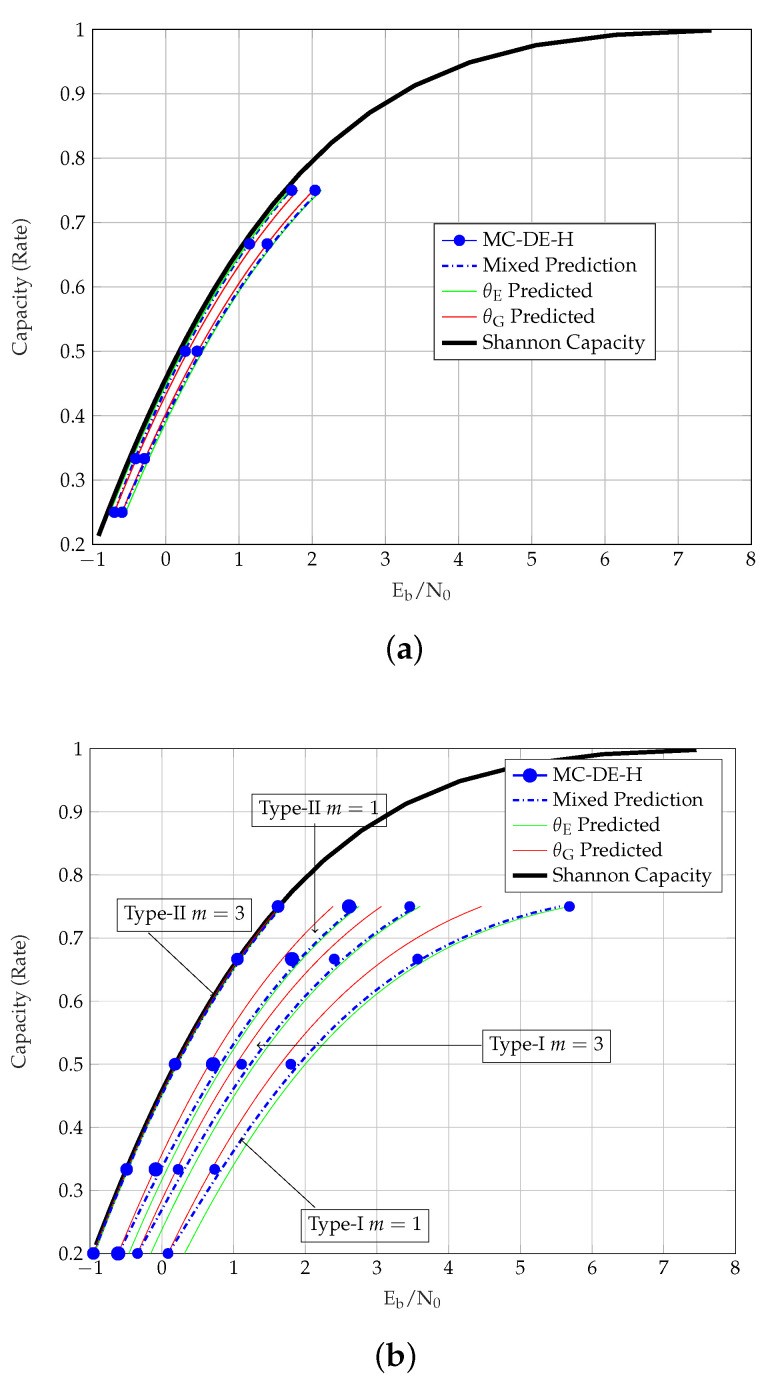
Thresholds of SC-SCCs and SC-HCCs over the AWGN channel. (**a**) $E_{b}/N_{0}$ vs Rate of SC-SCCs with m=1 and m=3. (**b**) $E_{b}/N_{0}$ vs Rate of type 1 and type 2 SC-HCCs-II with m=1 and m=3.

**Figure 5:**
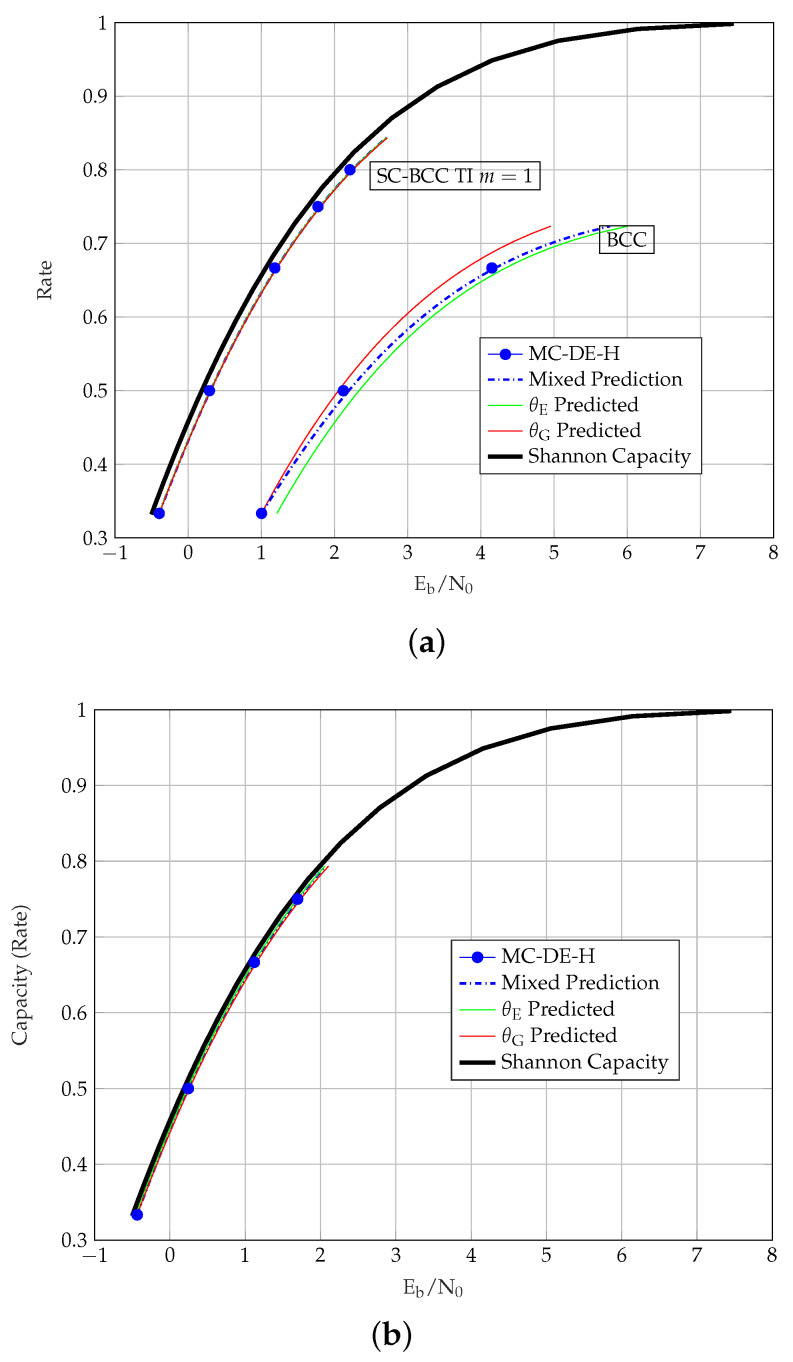
Thresholds of BCCs over the AWGN channel. (**a**) $E_{b}/N_{0}$ vs. Rate of BCCs and SC-BCCs with m=1. (**b**) $E_{b}/N_{0}$ vs. Rate of SC-BCCs with m=3.

From the thresholds of different TC ensembles, we also observe that the similarity of the MC-DE-H and the predicted thresholds depends on an ensemble’s strength. For stronger TCs, the similarity between the predicted and the MC-DE-H thresholds is clearly observed, as shown for m={1,3} SC-SCCs in Figure 4a, m={1,3} SC-BCCs in Figure 5, and m=3 SC-HCCs-II type-II in Figure 4b. We further observe that the predicted and the MC-DE-H thresholds are not alike (1) for PCCs in Figure 3, as these have BP thresholds close to the MAP thresholds but relatively poor MAP thresholds (2) for other uncoupled TCs in general and SC-HCCs-II type-I in Figure 4b, as these have strong MAP but poor BP thresholds and (3) for the SC-HCCs-II type-II with m=1 in Figure 4b, which have a good MAP threshold but require a larger coupling memory.

SC-HCCs-II offer an interesting insight regarding the similarity of predicted and MC-DE-H thresholds of an ensemble. HCCs-II have the strongest MAP thresholds on the BEC among all considered TC ensembles, and we expect the threshold predictions to show strong similarity with spatial coupling. From the thresholds of the SC-HCCs-II in Figure 4b, however, we observe that this strong similarity is only visible for the SC-HCC-II type-II ensemble with m=3. The SC-HCC-II type-I ensemble, which uses a different type of coupling than the type-II, shows a weaker BP performance and lower similarity than the type-II, even at a larger coupling memory m=3. This suggests that in addition to a strong MAP threshold, the capability of an ensemble to exhibit very similar predicted and MC-DE-H thresholds is also linked to its ability of achieving threshold saturation [14]. For those SC-TCs that demonstrate threshold saturation, we have additionally computed the binary symmetric channel (BSC) thresholds and compare the entropy of the thresholds for the BSC, BEC and AWGN channel in Table 5. A similar entropy *h* at the thresholds of the selected SC-TCs over all three channels confirms our conjecture that a strong similarity between predicted and MC-DE-H thresholds of an ensemble is associated with its capability of achieving threshold saturation.

**Figure 6:**
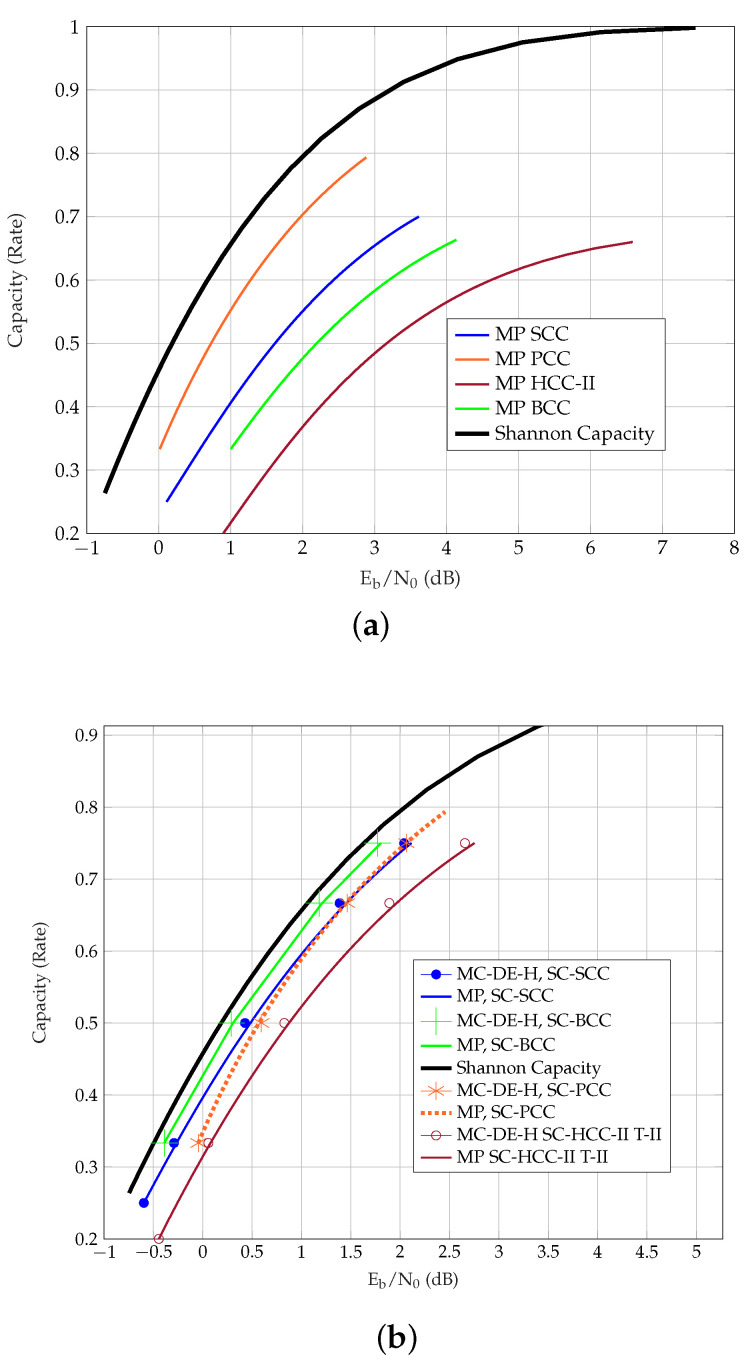
Comparison of TC ensembles in terms of the AWGN channel Thresholds. (**a**) $E_{b}/N_{0}$ vs. Rate of uncoupled TCs. (**b**) $E_{b}/N_{0}$ vs. Rate of SC-TCs with m=1.

In order to provide an overview over the different ensembles, the MC-DE-H and MP thresholds of uncoupled and coupled TCs with m=1 are plotted in Figure 6. For coupled TCs with m=1, BCCs perform best among the considered ensembles, whereas PCCs are the first among the uncoupled ensembles. Interestingly, SC-PCCs approach SC-HCCs at lower rates and SC-SCCs at larger rates. Note that the performance of SC-HCCs-II-TII can be improved by increasing the coupling memory and it is observed that they then outperform SC-BCCs with m={1,3}.

**Figure 7:**
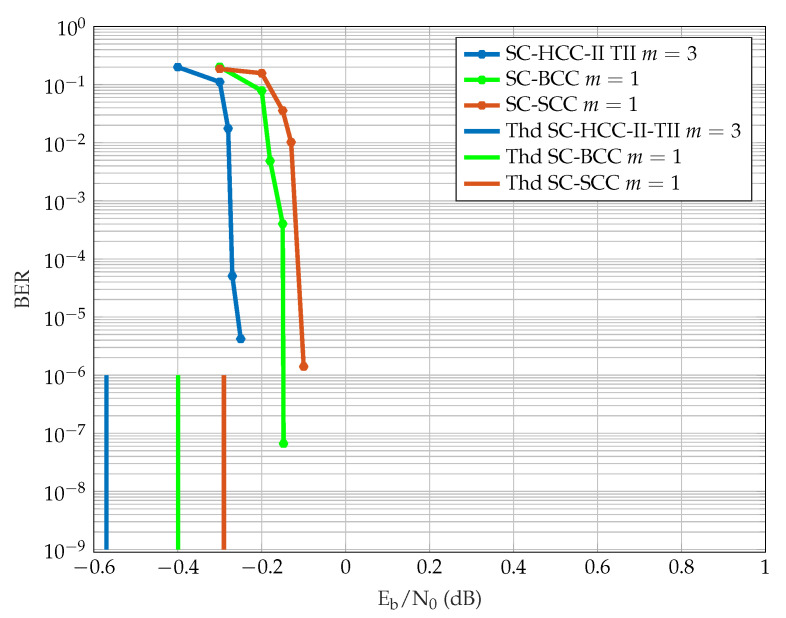
Finite block length performance of rate 1/3 ensembles with equal latency.

The threshold observations are further validated by performing some finite length BER performance simulations for SC-HCC-II type-II codes with m=3, and SC-SCC and SC-BCC codes with m=1 for equal rate R=1/3 and equal decoding latency. The SC-HCC-II type-II code with m=3 is chosen because the m=1 ensemble has a poor BP performance on both the BEC and the AWGN channel compared to SC-BCCs and SC-SCCs with m=1. We use sliding window decoding with a window size W=8, coupling length L=100, and 20 decoding iterations at each window position. The input block length is N=16,384 for all ensembles, resulting in an overall structural decoding latency of 3NW=393,216 code symbols. The BER performance results are shown in Figure 7. It is observed that the SC-HCC-II type-II code with m=3 has the best performance followed by the SC-BCC code and SC-SCC code with m=1 respectively. The simulations are observed to be consistent with the BP thresholds. However, the gain of the HCC ensemble in terms of the threshold is larger is than in terms of the simulated waterfall performance. This partially can be prescribed to the limited window size.

In Table 6, we list the BEC and the AWGN channel thresholds of the TCs along with the parameters $\theta_{E}$, $\theta_{G}$ and $\theta_{\mathrm{MP}}$. By using these values together with the prediction methods described in Section 4, it is possible to immediately reproduce all the continuous threshold curves in Figure 2, Figure 3, Figure 4, Figure 5 and Figure 6. The computation of MC-DE-H thresholds, which are shown as blue dots in these figures, is very time consuming and provided only for validation of the prediction methods. Consider the MP thresholds of rate 1/3 SCCs as an example to show how to calculate the predicted thresholds using Table 6. First, we obtain the noise threshold $\sigma^{*}=1.3963$ of rate R=1/4 SCCs from its MC-DE-H threshold $E_{b}/N_{0}\;\left(\mathrm{dB}\right)=0.1109$. Next, we apply the entropy at the noise threshold $h_{G}\left(1.3963\right)=1-C_{G}\left(1.3963\right)=0.7035$, and $\theta_{\mathrm{MP}}=1.2601$ from the Table 6 to (14) for R(α=1/4)=1/3. This gives us $h_{G}\left(\sigma_{BP}\left(\alpha =1/4\right)\right)=0.5985$. From the AWGN channel capacity $C_{G}\left(\sigma \right)=1-0.5985=0.4015$, we obtain $\sigma =C_{G}^{-1}\left(0.4015\right)=1.1461$. Lastly, by using that $\sigma^{2}=1/\left(2\cdot E_{b}/N_{0}\cdot R\left(\alpha \right)\right)$ we obtain the MP for rate R=1/3 SCCs in terms of $E_{b}/N_{0}\;\left(\mathrm{dB}\right)=0.5761$.

The prediction methods provide a convenient way of comparing the thresholds of mother code ensembles with different rates. A randomly punctured code ensemble is characterized by the parameter θ≥1, where θ=1 corresponds to a capacity achieving ensemble [11]. An ensemble with a smaller $\theta_{E}$ and $\theta_{G}$ will outperform an ensemble with larger $\theta_{E}$ and $\theta_{G}$ at all achievable rates.

## 6. Conclusions

In this paper, we have performed a BP decoding threshold analysis of SC-TCs on the AWGN channel and demonstrated that the prediction methods presented in [8,11] can be used to approximate the thresholds efficiently. The prediction methods approximate the AWGN channel thresholds of the considered ensembles in a computationally efficient manner by using their BEC thresholds. Conventionally, MC-DE or EXIT function analysis are applied to analyze thresholds of TCs over the AWGN channel. Although these methods can provide a very accurate estimate of the BP thresholds, they are computationally expensive, especially for spatially coupled ensembles. Our results show that the predicted thresholds are very close to the MC-DE thresholds for strong spatially coupled ensembles such as SC-SCCs, SC-BCCs and SC-HCCs-II. It is further conjectured that the similarity between the predictions and MC-DE is associated with the strength of an ensemble and its threshold saturation capability. For strong coupled ensembles, universality is observed from the entropy of their thresholds over the BEC, AWGN channel and BSC. For uncoupled ensembles with random puncturing, the predictions are improved with help of both the AWGN channel and BEC threshold of the mother code ensembles.

## Figures and Tables

**Table 1:** BP thresholds of uncoupled serially concatenated codes (SCCs) obtained by Monte-Carlo density evolution with Gaussian approximation (MC-DE-GA), Monte-Carlo density evolution with histogram (MC-DE-H), and erasure channel prediction (ECP).

Thresholds	Rate			
$E_{b}/N_{0}$ (dB)	1/4	1/3	1/2	2/3
MC-DE-GA	0.11	0.50	1.46	2.95
MC-DE-H	0.12	0.51	1.50	3.05
ECP	0.37	0.76	1.74	3.25

**Table 2:** Erasure-, $\theta_{E}$ predicted- and MC-DE-H thresholds of SCC ensembles.

	SCC			SC-SCC		
Rate	ϵ	$\theta_{E}$ ($E_{b}/N_{0}$)	MC-DE-H	$\epsilon_{\mathrm{SC}}$	$\theta_{E}$ ($E_{b}/N_{0}$)	MC-DE-H
1/4	0.6896	0.37	0.12	0.7379	−0.54	−0.59
1/3	0.5861	0.76	0.51	0.6505	−0.22	−0.29
1/2	0.3792	1.74	1.50	0.4758	0.51	0.43
2/3	0.1723	3.25	3.05	0.3011	1.48	1.39
3/4	0.0688	4.70	-	0.2137	2.13	2.05

**Table 3:** Comparison of $\theta_{G}$ and $\theta_{E}$ predicted thresholds of SCCs.

Thresholds	Rate					
	$E_{b}/N_{0}$ (dB)	1/4	1/3	1/2	2/3	3/4
$\theta_{E}$ Predicted	0.37	0.76	1.74	3.25	4.70	
$\theta_{G}$ Predicted	0.12	0.50	1.40	2.72	3.82	
MC-DE-H	0.12	0.51	1.50	3.05	-	

**Table 4:** $\theta_{E}$ predicted and MC-DE-H thresholds of coupled turbo-like codes (TCs).

Ensemble	*m*	Thresholds	Rate					
		$E_{b}/N_{0}$ (dB)	1/5	1/4	1/3	1/2	2/3	3/4
Shannon Capacity			−0.9637	−0.7942	−0.4952	0.1872	1.0597	1.6262
SC-SCC	1	$\theta_{E}$ Predicted	-	−0.54	−0.22	0.51	1.48	2.13
SC-SCC	1	MC-DE-H	-	−0.59	−0.29	0.43	1.39	2.05
SC-SCC	3	$\theta_{E}$ Predicted	-	−0.75	−0.45	0.24	1.12	1.70
SC-SCC	3	MC-DE-H	-	−0.70	−0.41	0.27	1.15	1.73
SC-PCC	1	$\theta_{E}$ Predicted	-	-	-0.30	0.42	1.35	1.98
SC-PCC	1	MC-DE-H	-	-	−0.04	0.60	1.47	2.07
SC-HCC-II type-II	1	$\theta_{E}$ Predicted	−0.45	-	0.08	0.87	1.97	2.75
SC-HCC-II type-II	1	MC-DE-H	−0.60	-	−0.08	0.72	1.82	2.62
SC-HCC-II type-II	3	$\theta_{E}$ Predicted	−0.93	-	−0.46	0.23	1.11	1.68
SC-HCC-II type-II	3	MC-DE-H	−0.95	-	−0.49	0.19	1.06	1.63
SC-BCC	1	$\theta_{E}$ Predicted	-	-	−0.39	0.31	1.21	1.81
SC-BCC	1	MC-DE-H			−0.39	0.30	1.19	1.78
SC-BCC	3	$\theta_{E}$ Predicted	-	-	−0.45	0.24	1.12	1.70
SC-BCC	3	MC-DE-H			−0.43	0.25	1.13	1.70

**Table 5:** Entropy *h* of TCs over the binary erasure channel (BEC), binary symmetric channel (BSC) and additive white Gaussian noise (AWGN) channel.

Ensemble	Rate	$h_{\mathrm{BEC}}$	$\epsilon_{\mathrm{BSC}}$	$h_{\mathrm{BSC}}$	$h_{\mathrm{AWGN}}$
SC-BCC m=3	1/3	0.6644	0.1718	0.6618	0.6630
SC-SCC m=3	1/4	0.7483	0.2114	0.7442	0.7456
SC-SCC m=3	1/3	0.6644	0.1708	0.6595	0.6616
SC-HCCII type-II m=3	1/5	0.7990	0.2427	0.7995	0.7996
SC-HCCII type-II m=3	1/3	0.6650	0.1738	0.6663	0.6664

**Table 6:** θ parameter of various prediction methods.

Ensemble	Rate	MC-DE-H	ϵ	Parameters		
		$E_{b}/N_{0}$ (dB)		$\theta_{E}$	$\theta_{G}$	$\theta_{\mathrm{MP}}$
PCC	1/3	0.02	0.6428	1.0716	1.0953	1.0597
SC-PCC, m=1	1/3	−0.04	0.6553	1.0341	1.0839	1.0092
SCC	1/4	0.12	0.6896	1.2416	1.1880	1.2595
SC-SCC, m=1	1/4	−0.59	0.7379	1.0484	1.0398	1.0513
SC-SCC, m=3	1/4	−0.70	0.7483	1.0068	1.0182	1.0030
HCC	1/5	0.90	0.7044	1.4780	1.4339	1.4890
SC-HCCII-TII, m=1	1/5	−0.60	0.7790	1.1050	1.0748	1.1125
SC-HCCII-TII, m=3	1/5	−0.95	0.7990	1.0050	1.0027	1.0056
BCC	1/3	1.01	0.5541	1.3377	1.2943	1.3594
SC-BCC, m=1	1/3	−0.39	0.6609	1.0173	1.0190	1.0165
SC-BCC, m=3	1/3	−0.43	0.6644	1.0068	1.0117	1.0043
